# Beyond the monorail perspective: constructing a dual-role engagement framework in peer feedback for second language oral communication

**DOI:** 10.3389/fpsyg.2026.1804403

**Published:** 2026-03-04

**Authors:** Wanning He, Xiaoyan Jin

**Affiliations:** 1 School of International Chinese Education, Northeast Normal University, Changchun, Jilin, China; 2 School of International Education, Liaoning University of Science and Technology, Anshan, Liaoning, China

**Keywords:** cooperative learning theory, dual-role framework, learner engagement, peer feedback, second language oral communication, sociocultural theory

## Abstract

This paper aims to examine and transcend the “single-track” analytical paradigm of peer feedback research in the field of second language acquisition (SLA), and construct a novel “Conceptual framework of dual-role engagement.” Traditionally, learner engagement research has primarily focused on learners’ cognitive, behavioral, and emotional responses as feedback recipients. Derived from research on teacher feedback and automated assessment systems, this perspective fails to fully capture the dual role of learners as both feedback providers and recipients/processors in peer feedback activities, as well as the interactive nature of their roles. This study pays particular attention to the context of second language oral communication, precisely because oral feedback is highly immediate, interactive, and can most vividly reflect learners’ dynamic engagement process in switching between dual roles. This paper first outlines the paradigm shift in feedback concepts from one-way transmission to dialogue and negotiation, as well as the core characteristics of peer feedback as a social and collaborative language practice. Subsequently, it systematically reviews the development trajectory of learner engagement theory and its application and limitations in SLA feedback research, pointing out the theoretical gaps when directly applying the classic three-dimensional (cognitive, behavioral, emotional) model to peer feedback contexts. Based on this, this study proposes a “dual-role engagement framework” specifically tailored for SLA oral peer feedback, supported by sociocultural theory and cooperative learning theory. This framework examines learners’ engagement simultaneously on two interrelated tracks: “providing feedback” and “processing feedback,” elaborating on the specific connotations, observational indicators, and interactive mechanisms of learners’ cognitive, behavioral, and affective engagement when playing different roles. This study serves as a conceptual proposal. It represents a refined development of learner engagement theory. It also provides new conceptual tools to advance peer feedback research. Specifically, these tools facilitate a shift from a “result-oriented” to a “process-oriented” approach, and from viewing feedback as one-way transmission to understanding it as dialogue construction. Future research can commence from four aspects: the development of measurement tools, the verification of dynamic mechanisms, the exploration of influencing factors, and the testing of practical applications, to empirically consolidate and refine the theoretical framework.

## 1 Introduction

In the context of a profound transformation in second language teaching paradigms, shifting from “teacher-centered” to “student-centered” and from “outcome-oriented” to “process-oriented,” peer feedback has evolved from a supplementary teaching technique into a core component for cultivating learners’ collaborative learning abilities, critical thinking, and language communication skills (Liu and Hansen, 2002; Xu, 2020). Especially in oral language teaching, peer feedback provides learners with opportunities for rich language practice and meaning construction beyond traditional teacher-student models by creating authentic interactive negotiation situations (Swain, 2006; Nicol, 2010).

### 1.1 The rationale for a dual-role focus in L2 oral peer feedback

This study selects second language (L2) oral communication as the context for constructing a dual-role engagement framework due to the distinctive research value of oral feedback. Dual-role engagement refers to learners’ simultaneous participation in peer feedback activities as both feedback providers and feedback receivers/processors.

Oral peer feedback is treated as a crucial research context because it closely aligns with the authentic communicative essence of language acquisition. Unlike written feedback, oral feedback directly simulates the immediate correction, turn-taking, and meaning negotiation processes of everyday conversation, thereby approximating the social-interactive nature of language use (Swain, 2006). Learners focus not only on linguistic forms but also on processing multimodal signals, including intonation, pauses, eye contact, and gestures, which are integral to communicative intent and pragmatic competence.

Oral feedback possesses three key characteristics. First, it is highly instantaneous and dynamic. Learners must complete the rapid cognitive-behavioral sequence of listening, diagnosing, and formulating feedback in real time, while simultaneously processing received feedback and revising subsequent output. This time pressure makes cognitive load, behavioral strategies, and emotional experiences significant and observable, potentially fostering deep “flow” engagement (Payant and Zuniga, 2022). Second, oral feedback is multimodal, involving paralinguistic and non-linguistic cues such as voice, tone, rhythm, gaze, and gestures that enrich interaction and support self- and co-regulation (Clayton Bernard and Kermarrec, 2022). Learners’ dual-role engagement necessarily encompasses perception and interpretation of these signals, creating more complex engagement patterns than written feedback. Third, oral proficiency as a multidimensional construct, encompassing fluency, pronunciation, grammatical complexity, and interactive communication, relies on practice and adjustment in real social interactions. Peer feedback creates a low-threat “social practice environment” where learners can expose problems, negotiate, and practice immediately, helping overcome L2 oral anxiety and cultivate communication confidence.

Exploring dual-role engagement in oral contexts reveals the dynamic and reciprocal nature of peer feedback and provides a critical lens for understanding L2 oral development through social interaction. Current research, while confirming overall effectiveness of peer feedback (Vuogan and Li, 2022) and bilateral benefits (Gao et al., 2023), largely focuses on receivers, leaving provider engagement underexplored. For example, prompt design influences feedback quality (Mu and Schunn, 2025), yet its relationship to provider engagement remains unclear. Research on oral feedback processes remains underdeveloped, lacking systematic frameworks to capture learners’ dynamic engagement in both providing and receiving feedback (Yu and Hu, 2017). Most studies rely on analytical tools designed for written feedback, failing to account for oral communication’s unique characteristics of immediacy, multimodality, and interactivity. Furthermore, oral peer feedback more readily elicits observable manifestations of affective and social engagement, such as anxiety, confidence, empathy, willingness to collaborate, which are more direct and measurable in face-to-face real-time interactions (Baralt et al., 2016). These factors influence feedback effectiveness and are indispensable dimensions of learner engagement. Thus, constructing a dual-role engagement framework tailored to oral contexts addresses both a theoretical necessity and an urgent pedagogical need to enhance oral interaction quality.

### 1.2 The research gap and this study

However, the current evaluation of peer feedback effectiveness remains largely constrained by an implicit “unilateral” analytical perspective, primarily stemming from research on teacher and automated feedback (Ellis, 2010; Han and Hyland, 2015). This recipient-focused framework, while valuable for understanding feedback uptake, implicitly treats the ability to provide feedback as either unproblematic or peripheral. Such a stance overlooks substantial evidence that diagnosing a peer’s work and formulating constructive comments constitute a powerful cognitive activity for the provider (Reinholz, 2016). By adopting this unilateral lens, research omits half of the interactive picture. Essentially, it treats learners as passive feedback “terminals,” overlooking their active role as generators and collaborators in peer feedback activities (Philp and Duchesne, 2016).

Consequently, the existing paradigm fails to systematically examine the complex and dynamic dual-role engagement exhibited by learners as both “feedback providers” and “feedback receivers.” Specifically, the cognitive effort learners invest as reviewers in diagnosing issues, the sense of responsibility they bring to the role, and how these “provider” experiences influence their subsequent learning as “receivers” have not yet received sufficient systematic scrutiny within current analytical frameworks (Gibbs, 2014; Dawson et al., 2019).

Recently, the concept of feedback literacy has emerged as a pivotal framework for understanding how learners engage with feedback processes (Carless and Boud, 2018). This perspective aligns closely with the dual-role engagement proposed in this study, as it implies that learners must develop capacities not only as receivers but also as providers of feedback (Banister, 2023). However, existing feedback literacy research has predominantly focused on the receiver’s role, leaving the provider’s engagement undertheorized. The present framework directly addresses this gap by conceptualizing feedback provision as an integral component of learners’ feedback literacy development.

The core purpose of this paper is to deconstruct and transcend the “single-track” engagement view in peer feedback research, and to construct a theoretical framework that can simultaneously accommodate and analyze learners’ dual role engagement. Firstly, this paper will elaborate on the modern shift of the concept of feedback and the interactive essence of peer feedback, establishing the theoretical premise for constructing a new framework. Secondly, it will systematically sort out the multidimensional connotations of the concept of learner engagement and its application in feedback research, and point out its analytical blind spots in peer feedback. On this basis, the core part of this paper will propose and elaborate on the “Dual-Role Engagement Framework for Peer Feedback in Second Language Oral Communication”, defining the specific components and interactive relationships of learners’ cognitive, behavioral, and affective engagement on the “feedback provision track” and “feedback processing track”. Finally, this paper will explore the theoretical contribution and practical implications of this framework for the context of second language oral teaching, and point out future research directions.

This study is conceptual in nature, aiming to construct a theoretical framework whose validity and practicality await verification through future empirical research. It is important to note that this framework is specifically tailored to the context of L2 oral peer feedback; its applicability to other feedback settings (e.g., written feedback or non-language subjects) would require careful contextual adaptation and empirical validation, as discussed in Section 4.3. It addresses two core research questions: (1) How can a theoretical framework be developed to simultaneously accommodate and analyze learners’ dual-role engagement, as both providers and receivers/processors, in peer feedback within second language oral communication? (2) What are the specific components, internal interactive mechanisms, and theoretical implications of this Dual-Role Engagement Framework, as well as its practical insights for understanding the development of second language oral proficiency?

## 2 Theoretical framework development

### 2.1 Literature review

#### 2.1.1 Development of feedback concept

The connotation of feedback in the field of education has undergone profound evolution. Early views tended to regard feedback as a one-way process of information transmission from knowledge holders (such as teachers) to learners (Hattie and Timperley, 2007). This definition of “feedback as information” focuses on the accuracy and clarity of feedback content, with its function being understood as narrowing the gap between learners’ current performance and desired goals (Sadler, 1989).

Over the past twenty years, the dominance of the transmission model in feedback has been significantly challenged. A profound rethinking of its core concept has emerged, driven by the principles of social constructivism and a greater emphasis on learner autonomy and social interaction. Central to this rethinking is the idea that feedback is fundamentally dialogic. As Askew and Lodge (2000) famously argued, effective feedback operates as a dialogue that fuels the learning process. This view transforms feedback from a linear transaction into a dynamic, two-way negotiation of meaning between the giver and receiver (Yang and Carless, 2013). Consequently, learners have moved from being passive receptacles of information to active agents who must interpret, question, and integrate feedback to construct their own understanding (Carless, 2006). Carless and Boud (2018) succinctly capture the evolution of this learner-centered paradigm in a key definition. They describe feedback as “the process in which learners understand and utilize information from various sources to improve the quality of their work and learning strategies.” This definition marks a pivotal turn in research priorities. It shifts the focus from the instructor’s act of delivering feedback to the learner’s critical role in processing and applying it. In essence, this perspective reframes feedback from a static product (like comments on a page) to an ongoing and learner-driven process. The “dialogic turn” in the evolution of feedback concepts provides a foundational premise for the interactive mechanisms emphasized in this framework. This dialogic view is further developed in Er et al.’s (2021) collaborative learning framework for dialogic peer feedback, which positions feedback as socially mediated dialogue. Carless and Winstone (2023) also highlight the reciprocal relationship between teacher and student feedback literacy, reinforcing feedback as co-constructed meaning-making.

#### 2.1.2 The interactive nature and dual role of peer feedback

As understandings of feedback have evolved, so too has the conceptualization of peer feedback, which is now recognized as a rich, multidimensional practice involving learners in critiquing each other’s work and offering suggestions for improvement (Liu and Hansen, 2002). Its learning potential stems from two intertwined characteristics: its inherently social, interactive nature and the unique duality of roles it entails. As a sociocultural practice, peer feedback transcends mere error correction to become a foundational mode of collaborative learning (Swain, 2006). Through “languaging,” engaging in joint discussion and feedback formulation, learners are propelled into higher-order cognitive processing and meaning-making, thereby creating a “dialogic space” (Nicol, 2010) wherein continuous negotiation occurs via questioning, clarification, explanation, and reformulation (Carless, 2006). Such interaction represents not superficial discussion of form but a deeper integration of perspectives (Hovardas et al., 2014), effectively providing “scaffolding” that helps learners advance through their zone of proximal development (Vygotsky, 1978).

More distinctively, peer feedback is defined by its required role duality: each participant simultaneously acts as feedback giver and receiver/processor (Gibbs, 2014). As givers, learners actively apply their knowledge and evaluative criteria to analyze peers’ work, thereby deepening their internal grasp of learning objectives (Han and Xu, 2019). As receivers, they interpret, critically evaluate, and decide on feedback, honing critical thinking and metacognitive skills. This constant role-switching cultivates audience awareness and, through sustained metalinguistic reflection, fosters a more profound understanding of language (Yang and Carless, 2022). Recent studies further underscore bidirectional influence: receivers’ questioning prompts providers to reflect (Zhu and To, 2022), engagement varies in depth across roles (Wu and Schunn, 2023), and proficiency moderates the benefits each role confers (Gao et al., 2023), collectively pointing to the need for a dual-role framework.

Moreover, multimodality constitutes a core feature of oral peer feedback. In L2 oral contexts, paralinguistic (e.g., intonation, pauses) and non-verbal resources (e.g., gaze, gestures) serve socio-affective functions such as managing face threats, conveying empathy, and coordinating turn-taking (Baralt et al., 2016). Researchers have begun examining multimodal mechanisms in peer feedback. Ke’s (2025) case study in video-conferencing English classrooms in Taiwan provides valuable empirical evidence: analyzing 46 instances of peer corrective feedback, the study found that providers extensively employed multimodal strategies, using smiles to mitigate face threat and gaze shifts to manage interactional pressure, thereby enhancing positive peer interaction. Stimulated recall interviews revealed that learners not only attended to linguistic content but also interpreted peers’ non-verbal cues to judge intent and build trust. The study also identified challenges in online environments, such as attenuation or distortion of non-verbal cues, highlighting the complexity of multimodal engagement across different media (online vs. offline, synchronous vs. asynchronous). Despite these insights, significant gaps remain: most peer feedback research still focuses on written feedback, lacking systematic description of multimodal interaction in oral feedback; moreover, when learner engagement theories (e.g., Fredricks et al., 2004) are applied to oral contexts, they fail to incorporate multimodal perception, production, and interpretation as independent dimensions. Therefore, any analytical framework aiming to comprehensively capture engagement in oral peer feedback must integrate multimodal elements as a core consideration.

In summary, contemporary feedback theory frames feedback as a dialogic, learner-centered process of social construction (Carless and Boud, 2018; Yang and Carless, 2013). Peer feedback epitomizes this view, leveraging role duality and interactive negotiation to propel language learning. Here, reciprocity, the bidirectional, mutually influential relationship between providers and receivers, is fundamental to the collaborative nature of the activity (Gibbs, 2014; Reinholz, 2016). Recognizing this essential nature provides a crucial foundation for critically examining existing research and building more effective analytical frameworks.

#### 2.1.3 Learner engagement framework

Learner engagement refers to a focused, active psychological state exhibited during learning tasks (Fredricks et al., 2004; Koltovskaia, 2020). The most influential framework, proposed by Fredricks et al. (2004), comprises three dimensions: cognitive, behavioral, and affective engagement. Cognitive engagement involves psychological investment in learning, including deep cognitive strategies and metacognitive monitoring. Behavioral engagement refers to explicit participation in academic activities, measurable through objective indicators such as task duration and interaction frequency (Dörnyei and Kormos, 2000). Affective engagement encompasses learners’ emotional responses, including interest, enthusiasm, anxiety, and boredom (Mercer, 2019). Additionally, scholars emphasize social engagement, characterized by reciprocity and willingness to collaborate (Lambert et al., 2017; Svalberg, 2009).

Hiver et al. (2021) argue that learner engagement is a dynamic, context-dependent, and socially embedded construct, necessitating contextualized reconceptualization when applying the classic model to specific pedagogical contexts. Hiver and Wu (2023) further propose a dynamic systems perspective, emphasizing that cognitive, behavioral, emotional, and social dimensions are continuously coupled and mutually influential during interaction. This perspective offers crucial theoretical insights for understanding the dynamic switching of dual roles in peer feedback: engagement as “providers” and “receivers” is not two isolated tasks, but a dynamic process where each role triggers and regulates the other in real-time interaction.

From the above perspectives, learner engagement is a complex concept. It integrates internal psychological processes, explicit behavioral manifestations, and sociocultural interactions. Its core elements typically encompass four aspects, as synthesized in Table 1.

**Table 1:** Core dimensions of learner engagement.

Dimension	Key characteristics	Representative scholars
Cognitive engagement	Refers to sustained attention, mental effort, and the use of self-regulation strategies during the learning process. In interactions, asking questions, exchanging ideas, providing explanations, or defending arguments are manifestations at the discourse level.	Philp and Duchesne (2016)
Behavioral engagement	Directly related to learners’ level of focus and effort, often measured through objective indicators such as task duration, number of produced words, and number of turns.	Dörnyei and Kormos (2000)
Affective engagement	Concerns learners’ emotional experiences during participation. Positive emotions (such as interest and enthusiasm) are indicators of engagement, while negative emotions (such as boredom and anxiety) may lead to alienation. Includes positive learning attitudes, autonomy, and feelings towards peer relationships.	Philp and Duchesne (2016), Mercer (2019), Svalberg (2009), and Baralt et al. (2016)
Social engagement	Emphasizes the collaborative nature of interactions, with core characteristics manifested as reciprocity, mutual benefit, and willingness to listen among peers. Learners in this state tend to initiate conversations proactively.	Lambert et al. (2017) and Svalberg (2009)

The learner engagement framework has been increasingly applied in L2 feedback research. Ellis (2010) first introduced “engagement” in feedback studies, defining it as learners’ responses to received feedback across cognitive, behavioral, and affective dimensions. Han and Hyland (2015) subsequently refined this framework for written feedback, extending cognitive engagement to include interpretation and integration, behavioral engagement to encompass modification behaviors, and affective engagement to cover dynamic emotional responses. Zhang and Hyland (2018) further developed an integrated model examining engagement with both teacher and automated feedback. However, applying this receiver-centric framework to peer feedback reveals a critical gap: the “neglect of the provider role” (Yang and Carless, 2022). While the classic three-dimensional structure retains universal applicability, its direct application to peer feedback, particularly oral contexts, requires reconfiguration. The present framework addresses this gap by mapping cognitive, behavioral, and affective dimensions onto both “provider” and “receiver” tracks, grounded in the “dual-role, dual-track” nature of peer feedback.

#### 2.1.4 Feedback literacy

Parallel to evolving feedback concepts, feedback literacy refers to the capabilities enabling learners to effectively participate in feedback processes (Carless and Boud, 2018). Carless and Boud (2018) defined it as “the understandings, capacities and dispositions needed to make sense of information and use it to enhance work or learning strategies.” Molloy et al. (2020) extended this with a learning-centered framework encompassing four features: appreciating feedback, making judgments, managing affect, and taking action, resonating with the tripartite engagement model adopted in this study.

Subsequent research has contextualized feedback literacy within peer feedback practices. Dong et al. (2023) developed and validated a scale assessing students’ peer feedback literacy in writing, identifying dimensions including providing constructive feedback, receiving feedback, and dialogic negotiation. Hoo et al. (2022) demonstrated that self- and peer-assessment interventions foster feedback literacy, emphasizing reciprocity in giving and receiving feedback. Banister (2023) explored peer feedback in academic and business English, highlighting meta-dialogues in developing feedback literacy, particularly relevant to this study’s focus on L2 oral communication. The affective dimension has been foregrounded by Bharuthram and van Heerden (2023), who found that both positive emotions (e.g., pride, satisfaction) and negative emotions (e.g., anxiety, discomfort) significantly influence learners’ engagement and feedback literacy development, underscoring the need to attend to affective experiences in both provider and receiver roles.

Feedback literacy is also shaped by contextual and cultural factors. Rovagnati et al. (2022) examined international postgraduate students, showing how feedback cultures and prior educational backgrounds impact feedback literacies, a factor that may moderate dual-role engagement in L2 oral peer feedback. Wood (2022) investigated technology-mediated dialogic peer feedback, demonstrating that structured online dialogues can enhance feedback uptake and literacy development, pointing to digital tools’ potential in scaffolding dual-role engagement. The interplay between teacher and student feedback literacy has been explored by Carless and Winstone (2023), who argue that teachers’ own feedback literacy crucially influences students’ development. Ketonen et al. (2020) provided longitudinal evidence that sustained peer feedback engagement fosters literacy growth, improving feedback quality and criterion understanding, while Hoo et al. (2022) showed that self- and peer assessment enhance evaluative judgment. Rovagnati et al. (2022) further emphasize that cultural background shapes affective and social engagement, highlighting the situated nature of dual-role participation.

In summary, feedback literacy research offers a rich foundation for understanding learner engagement with feedback. However, this literature has predominantly examined feedback literacy from the receiver’s perspective, leaving competencies required for effective feedback provision underexplored. The proposed dual-role engagement framework addresses this gap by specifying the cognitive, behavioral, and affective engagements constituting both providing and processing feedback, thereby enriching the theoretical grounding for feedback literacy development in L2 oral contexts.

### 2.2 Theoretical basis

The construction of this framework primarily relies on sociocultural theory and cooperative learning theory (Vygotsky, 1978; Slavin, 1980). Sociocultural theory emphasizes that learning occurs through social interaction and collaborative construction (Vygotsky, 1978). This theory provides a fundamental theoretical perspective for understanding human cognition and learning, especially the interaction and feedback in the process of second language acquisition (Mayer, 2014). Sociocultural theory posits that human learning and cognitive development are essentially socially constructed processes. Individuals do not acquire knowledge in isolation, but gradually achieve the transformation of psychological functions from the primary to the advanced level through participating in social interactions and using cultural artifacts such as language and symbols as intermediary tools (Vygotsky, 1978). In the field of second language acquisition, this means that language development itself is dynamically constructed through social interaction, and learners acquire language by actively participating in meaning negotiation during interactions (Ohta, 1995). This perspective fundamentally redefines learning from an individual’s internal cognitive activity to a collaborative practice occurring in a sociocultural context.

Several key concepts within sociocultural theory provide direct support for this study. First, the concept of “mediation and internalization” posits that language is the most important psychological mediation tool (Vygotsky, 1978; Lantolf and Thorne, 2006). In the context of peer feedback, the feedback language itself serves as a core mediator. It manifests in the “provider track” as the organization of comments and in the “receiver track” as the comprehension of suggestions. In peer feedback, the feedback language itself serves as a core mediator. Learners, whether as feedback providers (organizing comments) or receivers (understanding suggestions), actively utilize this tool. The process of internalization, which refers to the process by which individuals transform experiences from social interactions into internal psychological functions (Swain, 2006), explains the ultimate goal of peer feedback—whether providing or receiving feedback, the deep engagement ultimately leads to the internalization of language knowledge and social rules. The ultimate goal of deep engagement in both the “providing” and “receiving” processes is the internalization of language knowledge and social norms. Sociocultural theory also foregrounds meta-dialogues—discussions about the feedback process, which deepen feedback understanding and literacy (Banister, 2023). Additionally, affective engagement, as explored by Bharuthram and van Heerden (2023), reveals that emotions such as anxiety and pride significantly influence feedback uptake and participation in both roles. In the concept of “Zone of Proximal Development and Scaffolding”, Zone of Proximal Development (ZPD) refers to the gap between the actual level of problem-solving ability of learners independently and the potential level they can achieve with the assistance of others (Vygotsky, 1978). Scaffolding refers to the temporary support provided by more capable others (such as peers) to help learners cross the ZPD (Lee, 2017). In peer feedback, learners act as “experts” and “novices” to each other, working together within each other’s ZPD by providing “scaffolding” (i.e., feedback and negotiation) to each other. This strongly supports the “dual-role” framework, where providing feedback itself is a cognitive and affective engagement process of building scaffolding, and its value is no less than receiving and utilizing scaffolding. Existing research has largely understood “scaffolding” as the support that “receivers” obtain from “providers.” However, this framework emphasizes that the act of providing feedback is itself a cognitive and affective activity of scaffolding. When learners diagnose a peer’s problem and organize language to articulate suggestions, they are, in fact, constructing scaffolding for their own metacognitive abilities. This perspective unifies “teaching” and “learning” within the same agent, representing an extension of Vygotsky (1978) concept of “internalization.”

Sociocultural theory not only provides a philosophical foundation for understanding the interactive nature of peer feedback but also offers conceptual tools for analyzing the specific processes involved in the participation of dual roles. Lantolf and Poehner (2014), in their work Sociocultural Theory and the Pedagogical Imperative in L2 Education, emphasize that the core value of sociocultural theory lies in its “practicality”, that is, the need to transform theoretical concepts (such as mediation, internalization, and the zone of proximal development) into observable and operable pedagogical practices. The proposed framework is a practical embodiment of this principle. It concretizes the abstract concept of “scaffolding” into cognitive behaviors on the provider track, such as “in-depth analysis,” “standard application,” and “feedback construction,” while operationalizing the concept of “internalization” into cognitive operations on the receiver track, including “attention and decoding,” “evaluation and integration,” and “implementation of modifications.”

Cooperative learning, as a systematic teaching theory and strategy, has been the focus of research in the United States since the early 1970s. It has a rich theoretical foundation and development trajectory, and has had a profound impact on second language teaching. The theoretical basis of cooperative learning is deeply rooted in social constructivism. This perspective holds that knowledge is not acquired in isolation by individuals, but is constructed and developed through social interaction and collaboration (Bruffee, 1984). This view places the learning process at the core of social interaction, emphasizing that communication, feedback, and meaning negotiation among peers are important mechanisms for knowledge internalization and ability development. Within this theoretical framework, peer feedback is regarded as a typical practical form of cooperative learning, creating a beneficial social interaction environment. In this environment, learners can not only receive social support and assistance from peers (Hu and Lam, 2010), but also promote the development of various second language skills, including oral proficiency, through meaning negotiation in interaction.

Regarding the definition of cooperative learning, Slavin (1980) elaboration is representative. He defined cooperative learning as a classroom technique aimed at encouraging students to engage in various learning activities in small groups or teams, assisting each other, and jointly studying materials. The entire learning process places great emphasis on and relies on the active participation of each member as well as continuous communication and negotiation among members. Cooperative learning not only promotes cognitive development but is also crucial for cultivating students’ “groupness” and “sociality,” and is an effective way to enhance students’ socialization.

In the field of second language teaching, the concept and practice of cooperative learning have been widely applied, especially in language teaching (e.g., Wigglesworth and Storth, 2012). Many classroom teaching activities inherently embody the principles of cooperative learning. Cooperative learning theory posits that positive interdependence and promotive interaction are essential for collaborative learning (Johnson and Johnson, 2018). However, the theory has largely remained at the level of general principles, leaving the micro-interactional mechanisms underspecified. The dual-role engagement framework operationalizes these principles in the context of peer feedback by foregrounding role differentiation: the provider engages in diagnostic, evaluative, and scaffolding activities, while the receiver engages in attentional, interpretive, and integrative processes. Their interaction through structured negotiation embodies promotive interaction, while the mutual dependence between high-quality feedback and its uptake reflects positive interdependence. This role-sensitive operationalization thus extends cooperative learning theory into a fine-grained analytical tool for peer feedback research. Integrating peer feedback into oral language classrooms is a vivid embodiment of the application of cooperative learning theory. Firstly, this group-based activity subverts the traditional one-way error correction model dominated by teachers and passively accepted by students, shifting to an interactive model centered around students. In this model, learners have dual identities as both “teachers” and “students”: as correctors, they need to monitor their peers’ language output and provide feedback; as recipients of feedback, they need to accept and process the feedback from their peers, thereby noticing the gap between their interlanguage and the target language. This duality of roles greatly enhances learners’ metacognitive awareness and engagement. Cooperative learning theory provides further support for this framework from another dimension. As Johnson and Johnson (2018) emphasize in their work on cooperative learning, “providing feedback” and “receiving feedback” are not isolated, unidirectional acts but rather a bidirectional shaping process within a structure of positive interdependence. Feedback providers deepen their own language analytical abilities through the process of explaining, questioning, and revising others’ output, while receivers enhance the quality of their language production through absorbing, negotiating, and transforming feedback. Through promotive interaction, both parties achieve mutual growth under shared goals (mutual growth through reciprocal feedback). It is precisely this duality of roles and their immediate switching that prompts learners to examine issues from multiple perspectives, deepen their metacognitive awareness, and ultimately achieve deep learning. This perspective directly echoes the core proposition of this framework: in peer feedback, “providing feedback” and “receiving feedback” are equally important learning processes that mutually shape each other in interaction and jointly promote the development of language proficiency.

In summary, sociocultural theory demonstrates the social interaction nature of learning from a philosophical perspective, and provides rigorous theoretical concepts and analytical tools for analyzing learners’ multidimensional engagement in the dual role of providing and processing feedback through specific concepts such as mediation, internalization, zone of proximal development, scaffolding, and activity systems. Cooperative learning theory, from the perspective of social constructivism, establishes peer feedback as the core practical mechanism for collaborative knowledge construction. This theory explicitly states that learners assume both the roles of “teacher” and “student” in peer feedback. This dual role and its enhancement of metacognition and engagement directly form the theoretical basis for constructing the “dual-role engagement framework”.

### 2.3 Construction methods

As argued in Section 2.1.3, existing engagement frameworks applied to peer feedback have adopted a receiver-centric perspective, neglecting the provider’s role, a theoretical gap necessitating a dual-role framework capturing both engagement tracks. In a complete peer feedback cycle, learner engagement should be understood as a parallel, dual-track process. Track A (receiver track) involves engagement with received feedback, addressed by existing frameworks. Track B (provider track) encompasses engagement while providing feedback: cognitively applying evaluation criteria and organizing language; behaviorally using tools and initiating negotiation; emotionally experiencing responsibility, empathy, or anxiety (Reinholz, 2016; Miao et al., 2006). Berg (1999) training framework demonstrates that guiding providers to focus on higher-order issues enhances feedback constructiveness and writing quality. Provider engagement profoundly affects feedback quality, which influences receiver engagement and learning outcomes (Nicol et al., 2014). Technology-mediated studies further inform framework construction: Wood (2022) showed online platforms enhance feedback uptake and literacy; Mu and Schunn (2025) demonstrated prompt design affects feedback quality, yet its link to provider engagement remains underexplored, reinforcing the need for a dual-role perspective.

The framework construction proceeded through four steps. First, a critical literature review identified theoretical gaps: the classic three-dimensional engagement model (Fredricks et al., 2004) applied to peer feedback reveals a persistent unidirectional perspective viewing learners only as “receivers.” Second, theoretical foundations were applied, adopting sociocultural theory (Vygotsky, 1978; Lantolf and Thorne, 2006) and cooperative learning theory (Gibbs, 2014; Reinholz, 2016; Carless, 2006; Nicol, 2010), providing groundwork for understanding dual roles in providing and processing feedback. Third, dimensions were mapped and refined: retaining the classic cognitive, behavioral, affective structure, the framework maps these distinctly onto “feedback provision” and “feedback processing” tracks, refining connotations according to each role’s tasks. Fourth, interaction mechanisms were introduced, clarifying dynamic mutual shaping between tracks and reflecting peer feedback’s dialogic, socially constructed nature.

In summary, while the three-dimensional engagement framework is widely recognized, its application to peer feedback reveals insufficient attention to learners as feedback providers. Informed by this theoretical review, the present dual-track framework inherits the classic structure but applies it to two fundamental aspects: feedback provision (examining engagement as evaluators) and feedback processing and revision (examining engagement as authors). Behavioral engagement encompasses observable actions, providing written feedback on peer oral production, discussing feedback, revising one’s own production. Affective engagement covers attitudes and emotional reactions throughout listening, providing, receiving, and revising. Cognitive engagement denotes depth of understanding and analysis of peer oral production, along with cognitive and metacognitive strategies employed.

Ultimately, by elucidating dual-track, mutual shaping mechanisms across providing and processing feedback, this framework re-conceptualizes learners as active negotiators of meaning and co-constructors of knowledge, unlocking peer interaction’s potential to foster both language proficiency and collaborative competence.

## 3 The proposed dual-role engagement framework

While previous research has recognized learners’ dual roles in peer feedback (Gibbs, 2014) and noted that focusing solely on the “receiver” role means “observing only half of the conversation” (Philp and Duchesne, 2016), a systematic analytical framework capturing dynamic dual-role engagement and interactive mechanisms has yet to be developed. Earlier “dual-role” studies often stopped at acknowledging role duality. In contrast, this framework systematically reveals the specific composition, observable indicators, and inherent differences in learners’ cognitive, behavioral, and affective engagement across these two distinct roles. Regarding role interaction, earlier models tended to treat the two roles as static and parallel tasks. This framework’s core breakthrough lies in its dual-track interaction mechanism, emphasizing that engagement under both roles mutually influences and shapes each other in real-time interaction, transcending mere enumeration of role-based tasks. Furthermore, this framework is specifically constructed for second language oral peer feedback, serving as both a theoretical model and a contextualized analytical tool grounded in oral interaction characteristics.

Based on a re-examination of peer feedback interaction and limitations of existing engagement frameworks, this study proposes the “Dual-Role Engagement Framework for Peer Feedback in L2 Speaking” to comprehensively capture learners’ complex and dynamic engagement states resulting from role switching during peer feedback activities.

### 3.1 Framework composition

**Figure 1:**
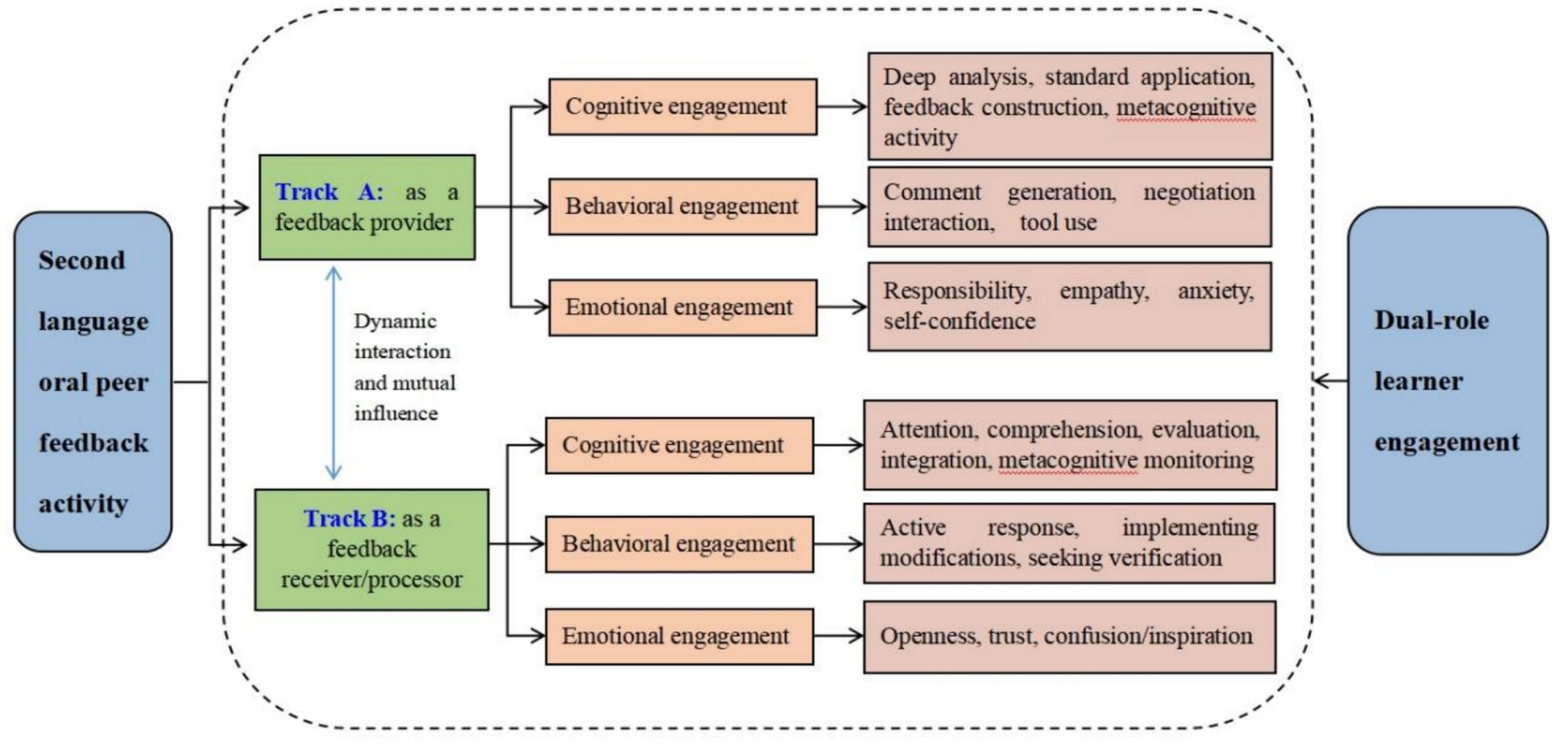
Dual-role engagement conceptual framework for peer feedback in second language oral communication.

The core concept of this framework is that in peer feedback, the total engagement of learners is the sum of their engagement as “providers” and their engagement as “receivers/processors,” and both influence and shape each other in the interaction. Neglecting either side will lead to an incomplete understanding of the feedback process and its effects. While previous research has acknowledged learners’ dual roles in peer feedback (Gibbs, 2014), such recognition has largely remained at the level of role identification, without systematically unpacking the multidimensional engagements entailed by each role. The present framework moves beyond this descriptive stance by operationalizing dual-role engagement. It specifies the cognitive, behavioral, and affective engagements that characterize learners’ participation as feedback providers (Track A) and as feedback receivers/processors (Track B). This role-sensitive operationalization enables researchers to move from asking “whether learners engage” to “how they engage across roles, with what depth, and with what observable indicators.” This framework consists of two parallel “engagement tracks,” each containing the three classic dimensions of cognition, behavior, and emotion, but their specific connotations vary greatly depending on the role (Figure 1). This figure serves as a heuristic and analytical model, illustrating the structural dimensions of dual-track engagement. It is designed to provide a structured conceptual tool for understanding and analyzing the peer feedback process, with its dynamic interactive relationships elaborated in detail below. The specific propositions and pathways remain subject to verification through future empirical research.

Figure 1 presents the two parallel tracks (feedback providing track A and feedback processing track B) of the dual-role participation framework and their constituent elements in a static and structured manner, aiming to provide readers with a clear conceptual framework. It is worth noting that Figure 1 presents an analytical and structured perspective, while Section 3.2 further explains the dynamic interweaving and mutual shaping process of these two tracks in actual interactions, with the two complementing each other. Figure 1 provides a basis for understanding the role division and participation dimensions in interactions, while Section 3.2 reveals how these dimensions interact and influence each other in real-time dialogue, negotiation, and revision, collectively constituting a circular learning system.

Track A captures learners’ engagement as feedback providers. Cognitive engagement on Track A unfolds through a multi-stage analytical process. The first stage is deep analysis, where the provider carefully listens to and dissects the peer’s oral production to identify strengths or issues in language form, content logic, and pragmatic appropriateness. Next, in standard application, the learner invokes learned knowledge, scoring criteria, or teacher-provided guidelines to evaluate the performance. Building on this, feedback construction entails organizing clear, specific, and constructive language. This translates internal judgments into understandable comments, often supported by examples or suggested modifications (Min, 2006). Throughout this process, metacognitive activity monitors the fairness and comprehensiveness of the evaluation. It also involves reflecting on whether the feedback is accessible to the receiver. In terms of behavioral engagement, learners demonstrate comment generation by investing time and effort in writing or preparing oral feedback points. This leads to negotiation interaction, where they actively initiate or join dialogue through questioning, clarification, and explanation to engage in deeper negotiation with peers (Nicol, 2010). Importantly, in oral contexts, behavioral engagement also manifests through multimodal strategies. Providers strategically employ non-verbal resources to support feedback delivery. For instance, when offering potentially face-threatening corrections, they may accompany their words with smiles, nods, or soft eye contact to soften criticism and convey empathy and support (Ke, 2025). They may also use gestures to simulate language problems or direct gaze to guide peers’ attention to specific expressions. The process is often supported by tool use, such as employing feedback checklists, scoring sheets, or recording playback to structure and enhance the feedback. Emotionally, engagement encompasses responsibility, an awareness of how one’s feedback influences peer learning; and empathy, the ability to offer constructive, peer-sensitive suggestions. It must be emphasized that empathy as an inner emotion needs to be perceived by receivers through explicit multimodal behaviors, such as attentive gaze, patient posture, and encouraging tone of voice to truly function as a positive affective force in interaction (Ke, 2025). These are often accompanied by self-confidence or anxiety, reflecting learners’ assurance in their ability to provide useful feedback or concerns about their evaluative competence.

Track B corresponds to learners’ engagement as feedback receivers and processors. Cognitively, engagement starts with attention and decoding, actively attending to and interpreting the literal and implied meanings of peer comments. This decoding process in oral feedback extends beyond linguistic content: receivers must invest cognitive resources in interpreting paralinguistic cues (e.g., intonation contours, pause lengths) and non-verbal cues (e.g., facial expressions, gaze direction) from their peers (Ke, 2025). Accurate interpretation of these multimodal signals is crucial for discerning the nature of the feedback (e.g., suggestion vs. command), its intent (helpful vs. critical), and its urgency. This is followed by evaluation and judgment, a critical assessment of the feedback’s relevance, accuracy, and utility, culminating in a decision on whether and how to adopt it (Yu and Hu, 2017). Subsequently, integration and reconstruction involve blending the feedback with self-assessment to revise or reformulate the original oral plan. Underpinning these steps is metacognitive monitoring, through which learners reflect on error causes, plan future improvements, and oversee the modification process. Behaviorally, engagement is expressed through active response, interacting with the provider by asking questions, requesting examples, or voicing doubts to clarify the feedback. In face-to-face interaction, receivers may also respond non-verbally (e.g., a confused expression, nodding to indicate understanding, or gaze seeking clarification), all of which constitute important forms of behavioral engagement that shape the ongoing dialogue (Ke, 2025). This may lead to implementing modifications, where learners adjust their language output in subsequent retellings or revisions based on the negotiated suggestions. At times, they may also engage in seeking verification, consulting teachers or other peers to confirm the accuracy of the feedback received. Affectively, engagement is characterized by openness and willingness to accept, a receptive attitude toward critique from peers, and by trust in peers’ intentions and competence, which shapes the degree to which feedback is internalized (Tsui and Ng, 2000). These affective states are closely tied to the interpretation of peers’ multimodal behavior: a genuine smile or attentive gaze can rapidly build trust, reduce defensiveness, and enhance openness to feedback; conversely, averted gaze or brusque tone may trigger anxiety, confusion, or resistance. These dispositions interact with immediate emotional reactions, which can range from feeling inspired and grateful to experiencing frustration, confusion, or defensiveness.

To clarify the dual-track engagement mechanism, consider the following example from an ESL classroom. During a speaking task titled “A Memorable Learning Experience,” Students A and B form a feedback pair. Student A first delivers an oral narrative. Afterwards, Student B consults a structured feedback form and writes a comment suggesting that the phrase “I have learned a lot when I worked there” could be improved to “I learned a lot while I was working there” to more accurately convey a past ongoing action. This initiates Track A, the feedback provision track, where B engages cognitively in linguistic diagnosis and behaviorally in generating written feedback. The pair then engages in oral negotiation. B explains the suggestion, focusing on the use of the past continuous tense. A responds by seeking confirmation, asking, “So, to show it was happening over time, I should say ‘I was working’?” This activates Track B, the feedback processing track, where A engages cognitively in understanding the feedback and behaviorally in active questioning. Subsequently, A revises the statement in a retelling, saying, “I learned a lot while I was working there last summer,” and notes the change in a written reflection. This shows a deepening of Track B through cognitive integration and behavioral modification. The instructor can assess engagement by analyzing B’s written feedback form for Track A quality, the negotiation dialogue for interactive performance, and a comparison of A’s initial and revised narratives for Track B improvement. During the negotiation phase, A asked, “So, I should use ‘was working’ to show it was ongoing?” (Track B cognition: decoding; behavior: active response). B immediately provided an example: “For instance, if you say ‘I was reading when she came in,’ the action of reading is ongoing.” (Track A cognition: standard application; behavior: elaborating explanation). A nodded and took notes upon hearing this, and subsequently used the past continuous tense correctly in their revision (Track B behavior: implementing modifications). In a post-activity interview, B reflected, “While helping A analyze this, I suddenly realized that I sometimes mix up past tenses too. I need to pay attention to that next time.” (Track A—Track B cross-track transfer: the provider’s metacognitive reflection). B also reflects on affective engagement in Track A, mentioning an effort to use collaborative language like “Maybe we can try...” to sound supportive. A reflects on affective engagement in Track B, stating that the discussion clarified the grammar point and that successfully revising the sentence boosted confidence.

The dual-role engagement framework aligns closely with feedback literacy. Track A engagements (e.g., deep analysis, feedback construction, empathy) reflect what Dong et al. (2023) term “providing constructive feedback” and Molloy et al.'s (2020) “making judgments” and “managing affect.” Track B engagements (e.g., attention and decoding, evaluation, openness) correspond to “appreciating feedback” and “taking action” (Molloy et al., 2020). By integrating both tracks, our framework offers a role-sensitive operationalization of feedback literacy in L2 oral peer feedback. As Ketonen et al. (2020) demonstrated, sustained peer feedback engagement fosters feedback literacy development, and our framework provides a granular lens for examining this process across dual roles. Moreover, the teacher’s role in scaffolding this development (Carless and Winstone, 2023) can be conceptualized as facilitating both Track A and Track B engagements through targeted guidance. In second language oral communication, the switching between dual roles imposes significant cognitive load on learners. Unlike written feedback, oral feedback requires learners to simultaneously complete multiple cognitive tasks within seconds: as providers, they must listen, diagnose, formulate, and articulate feedback; as receivers, they must comprehend, evaluate, and prepare to respond. Johnson and Abdi Tabari (2022) point out that this real-time dual-task processing substantially increases cognitive burden, requiring learners to allocate attention between content regulation and interaction management, while coordinating metacognitive monitoring and emotional regulation. Therefore, the cognitive complexity of oral peer feedback underscores the necessity of constructing a dual-role engagement framework.

The specific manifestations of affective engagement across the two tracks are also closely tied to the interactive context. Dao and Sato (2021) dynamic tracking study of emotional states in second language interaction found that learners’ enjoyment and anxiety fluctuate continuously throughout interaction and are closely associated with specific interactive events, such as successful expression, receiving feedback, or gaining recognition. This finding corroborates this framework’s emphasis on affective engagement while also suggesting that researchers need to employ dynamic, process-sensitive tools, such as stimulated recall and real-time emotional reports, to measure affective engagement, rather than relying solely on retrospective questionnaires.

### 3.2 Dual-track interaction mechanism

This framework emphasizes that the engagement of learners on both tracks is not isolated, but rather involves continuous and dynamic interaction and mutual influence. Unlike linear process models that depict feedback as a unidirectional flow from provider to receiver, the dual-track interaction mechanism conceptualizes peer feedback as a dynamically coupled system. Track A (provision) and Track B (processing) unfold simultaneously in real-time dialogue, continuously shaping each other. The receiver’s active questioning (Track B) may prompt the provider to refine their explanation (Track A), while the provider’s empathetic delivery (Track A) may enhance the receiver’s openness and trust (Track B). This bidirectional, mutually shaping process constitutes the core of feedback as socially mediated learning. Empirical support for mutual shaping comes from Zhu and To (2022), who found that receivers’ active engagement prompts providers to reflect, validating bidirectional influence. Gao et al. (2023) further suggest that language proficiency may moderate this interaction, with lower-proficiency learners benefiting more from providing feedback.

Firstly, the influence from the provider to the receiver is reflected in the depth of the provider’s engagement (such as whether it has been carefully analyzed) directly affecting the quality of feedback, which in turn affects the receiver’s cognitive and affective engagement (such as whether they perceive the feedback as useful and whether they are willing to adopt it). The empathy (emotional engagement) demonstrated by the provider during feedback can increase the receiver’s trust and openness. Secondly, the influence from the receiver to the provider is manifested in the receiver’s positive response in negotiation (behavioral engagement), which can motivate the provider to explain their viewpoint more deeply (increasing cognitive and behavioral engagement). The receiver’s recognition and adoption of feedback brings a sense of achievement and efficacy to the provider (positive emotional engagement). The experience of engagement in one role will be internalized as knowledge or attitude, affecting engagement in another role. For example, deep consideration of evaluation criteria as a provider (cognitive engagement) will enhance the accuracy of self-assessment as a receiver. The two tracks can be temporarily separated analytically for descriptive purposes, yet in actual peer feedback activities, they occur simultaneously, interact continuously, and mutually shape each other. Interaction may take place “immediately after feedback is provided,” “persist throughout the negotiation phase,” or be “indirectly reflected during the revision stage”; it can be quantified using indicators such as “the specificity of feedback entries” and “the number of negotiation rounds.” This dynamic may be moderated by factors such as learners’ language proficiency, task type, interpersonal relationships, cultural background, and the nature of teacher intervention.

The role of Track A (Feedback Provider) is essentially to construct “scaffolding” for peers. Cognitive and behavioral engagement such as “in-depth analysis,” “application of standards,” and “constructive feedback” constitute the specific content and process of building this scaffolding. The role of Track B (Feedback Receiver/Processor), in turn, is to utilize this scaffolding in an attempt to traverse their Zone of Proximal Development (ZPD). Cognitive engagement such as “attention and decoding,” “evaluation and integration,” along with behavioral engagement like “active response” and “implementation of revisions,” reflect their exploration and practice of higher-level language abilities with peer support. The dynamic interactive mechanism between the two tracks captures the real-time negotiation and adjustment process in the construction and use of scaffolding. The receiver’s confusion or questions (Track B) may prompt the provider to adjust their scaffolding (e.g., simplifying explanations, adding examples), while the quality of the provider’s feedback (Track A) directly influences the extent to which the receiver can utilize this scaffolding. This interactive process itself embodies cooperative development within the ZPD.

Based on the proposed dual-track interaction mechanism, future empirical research can test several hypotheses derived from this framework. We present a hypothetical scenario-based deduction in tabular form to illustrate the trajectory of dual-role engagement across different interactive contexts. The specific meanings of the hypothesis numbers (e.g., H1a, H2a, etc.) will be elaborated in detail later (see “3.4”) (Table 2).

**Table 2:** Hypothetical scenarios of dual-role engagement trajectories in peer interaction.

Context	Provider engagement (Track A)	Receiver response (Track B)	Interactive outcome	Hypothesized outcome
Scenario 1	High cognitive diagnosis + High affective empathy	High uptake + Active negotiation	Oral proficiency of both parties improves	Supports H1a, H2a
Scenario 2	High cognitive diagnosis + Low affective empathy	Low trust + Passive acceptance	Receiver revision limited; Provider’s sense of efficacy decreases	Supports H1b, H2b
Scenario 3	Low cognitive diagnosis + High affective empathy	High trust but limited revision	Positive affective atmosphere but limited learning outcomes	Requires further testing

### 3.3 Framework innovations

The dual-role engagement framework proposed in this study moves beyond merely acknowledging that learners play dual roles in peer feedback, a point already recognized in the literature (Gibbs, 2014). Instead, it offers a systematic and operational analytical lens that captures how learners engage cognitively, behaviorally, and affectively when assuming these roles, and how these engagements dynamically interact in real time. In doing so, the framework introduces three interrelated innovations that collectively reconceptualize peer feedback as a socially mediated learning process.

First, the framework establishes a dual-track analytical logic that is fundamentally different from the receiver-centric models derived from teacher or automated feedback research. While previous engagement frameworks (e.g., Ellis, 2010; Han and Hyland, 2015) have been valuable for understanding how learners process received feedback, they implicitly treat the provider’s role as either unproblematic or peripheral. By contrast, our framework places the provider’s engagement, diagnostic reasoning, feedback construction, metacognitive monitoring, and affective experiences such as empathy and responsibility, on equal footing with the receiver’s. This is not a mere mechanical superposition of two roles; rather, it reflects the inherent asymmetry of cognitive tasks and emotional demands between providing and processing feedback. The dual-track structure thus enables researchers to ask not only “How do learners respond to feedback?” but also “How do learners generate feedback, and what do they learn from that generative process?”

Second, the framework theorizes a dynamic mutual-shaping mechanism between the two tracks, transforming the static “role × dimension” grid into a cyclical system of co-construction. Unlike linear process models that depict feedback as flowing unidirectionally from provider to receiver, our framework conceptualizes peer feedback as a coupled system in which Track A (provision) and Track B (processing) continuously influence each other through real-time dialogue. For instance, a receiver’s puzzled gaze (a Track B behavioral cue) may instantly prompt the provider to elaborate or rephrase their feedback (Track A cognitive and behavioral adjustment), while a provider’s empathetic tone (Track A affective engagement) can enhance the receiver’s trust and openness (Track B affective response). This bidirectional, moment-to-moment shaping captures the essence of feedback as a dialogic and socially mediated activity, a dimension that remains invisible in traditional product-oriented analyses.

Third, and most distinctively, the framework is specifically calibrated for the unique affordances and constraints of L2 oral communication. Unlike written feedback, which allows for delayed reflection and revision, oral peer feedback unfolds under intense time pressure and relies heavily on multimodal cues (e.g., intonation, gaze, gesture). Our framework explicitly incorporates these features into its engagement indicators: (a) Immediacy is reflected in the rapid cognitive-behavioral sequences required of both providers (listening-diagnosing-formulating in seconds) and receivers (decoding-evaluating-responding in the next turn). (b) Multimodality is integrated into both tracks: providers use paralinguistic and non-verbal resources to soften criticism or signal support, while receivers must interpret these cues to accurately gauge the feedback’s intent and emotional tone. The framework thus provides a fine-grained tool for analyzing how multimodal signals mediate engagement and co-regulation (Ke, 2025). (c) Interactive negotiation becomes empirically tractable through indicators such as turn-taking patterns, clarification requests, and the use of mitigating strategies—phenomena that are central to oral interaction but often marginalized in written-feedback models.

By grounding these dimensions in oral interaction, the framework not only adapts generic engagement theory to a specific context but also reveals how the very nature of the medium (oral vs. written) shapes the engagement process. This contextualization sets a precedent for future research to similarly tailor engagement frameworks to other modalities (e.g., online asynchronous, video-conferencing).

In sum, the dual-role engagement framework transcends the descriptive recognition of role duality by providing an operationalizable, dynamic, and context-sensitive analytical apparatus. It shifts the research focus from “whether learners engage” to “how they engage across roles, with what depth, and under what conditions,” thereby unlocking the full explanatory potential of peer feedback as a collaborative learning practice.

### 3.4 Testable research hypotheses and operational indicators

Based on the theoretical construction of the dual-track interaction mechanism, this section translates the framework into testable hypotheses following a progressive logic from provider engagement to receiver response, receiver response to provider re-engagement, and dual-track interaction quality to learning outcomes, while incorporating moderating variables.

First, regarding provider influence on receiver response: the depth of the provider’s cognitive engagement in Track A (diagnostic accuracy, criteria application, feedback construction) positively predicts the receiver’s cognitive engagement in Track B (feedback comprehension, willingness to uptake, integration and reconstruction) (H1a). Concurrently, the provider’s affective engagement in Track A (empathy, responsibility) positively predicts the receiver’s affective responses in Track B (trust, openness), subsequently influencing revision behaviors (H1b). These hypotheses reveal that provider engagement quality stimulates the receiver’s deep processing and affective identification.

Second, concerning receiver influence on provider re-engagement: the receiver’s active response behaviors during negotiation (questioning, clarifying, challenging) positively promote the provider’s cognitive engagement in subsequent interaction, manifested as increased explanation depth and example use (H2a). The extent of the receiver’s feedback uptake positively influences the provider’s self-efficacy and responsibility, affecting their future engagement quality as a provider (H2b). These hypotheses reflect the bidirectionality and cyclicality of dual-track interaction.

Third, regarding dual-track interaction quality and learning outcomes: the depth of negotiation in dual-track interaction (number of interaction turns, frequency of mutual adjustments) positively predicts the quality of the receiver’s oral revisions (linguistic accuracy, complexity, coherence) (H3a). The affective climate within dual-track interaction (supportive language, positive non-verbal cues) positively predicts both parties’ oral fluency and complexity in subsequent tasks (H3b). These hypotheses link interaction process features to learning outcomes.

Finally, regarding moderating variables: learners’ language proficiency moderates H1a, with lower-proficiency learners relying more on provider cognitive engagement, while higher-proficiency learners are more susceptible to provider affective engagement (H4a). Cultural background (e.g., face concerns) moderates H1b: in face-conscious cultural contexts, provider empathy more strongly impacts receiver trust, though providers may experience higher anxiety when delivering feedback (H4b). These moderating variables enhance the framework’s adaptability to diverse learning contexts and support cross-cultural teaching research.

To enhance the framework’s transparency and facilitate empirical validation of the above hypotheses, Table 3 operationalizes each dimension across the two tracks by specifying observable indicators and suggested measurement methods. This table serves as a practical guide for researchers seeking to design coding schemes, interview protocols, or questionnaires that capture the multifaceted nature of dual-role engagement.

**Table 3:** Operational indicators and measurement methods for dual-role engagement.

Track	Dimension	Operational definition	Observable indicators	Measurement examples
Track A: provider	Cognitive	Mental effort in analyzing peer output, applying criteria, constructing feedback, and metacognitive monitoring	Accuracy of problem diagnosis Specificity of suggestions Use of evaluation criteria (e.g., rubric references) Self-corrections or hedging in feedback language	Content analysis: count of diagnostic statements, alignment with rubric - Stimulated recall: probe reasoning during feedback writing
	Behavioral	Observable actions in generating and delivering feedback	Length/detail of written comments Number of feedback points Use of tools (checklists, recordings) Initiating negotiation or clarification	Time spent on feedback Turn-taking frequency during negotiation Discourse analysis: speech act types (e.g., suggestions, questions)
	Affective	Emotional responses while providing feedback	Empathy markers (hedging, positive framing) Expressions of responsibility (“I think it’s important to…”) Non-verbal cues (nodding, smiling) Self-reported confidence/anxiety	Questionnaire items (e.g., “I felt responsible for helping my partner”) Video-based affect coding Post-task interviews
Track B: Processor	Cognitive	Mental processing of received feedback: attention, interpretation, evaluation, integration, metacognitive reflection	Number of clarification questions Accuracy of understanding (paraphrasing feedback) Evidence of comparing feedback with own judgment Reflection on errors and revision plans	Stimulated recall: recall thoughts when hearing feedback Revision notes analysis: what was accepted/rejected and why Think-aloud protocols during revision
	Behavioral	Observable responses to feedback: questioning, revising, seeking verification	Asking for clarification/elaboration Making revisions in subsequent output Consulting external resources (teacher, dictionary)	Count of uptake moves in revised speech Negotiation turns initiated Observation of revision behaviors
	Affective	Emotional reactions to receiving feedback: openness, trust, defensiveness, gratitude	Verbal expressions (“That’s helpful,” “I’m not sure I agree”) Non-verbal signs (frowning, nodding) Self-reported feelings	Affect questionnaire (e.g., “I felt anxious when receiving criticism”) Discourse analysis: emotional tone markers *Post-hoc* interviews

This operationalization enables researchers to move beyond theoretical abstraction toward systematic empirical investigation of dual-role engagement dynamics. The above hypotheses can be tested through various methods, including laboratory observation, classroom tracking, questionnaire measurement, and stimulated recall interviews. We recommend that future research adopt mixed-method designs, combining quantitative indicators (e.g., number of negotiation turns, revision amount, affective scale scores) with qualitative data (e.g., interactive discourse analysis, learner reflection records) to comprehensively capture the dynamic mechanisms of dual-role engagement and to validate the explanatory power and practical applicability of this framework in L2 oral instruction.

## 4 Discussion

### 4.1 Theoretical value

The proposed framework addresses a significant theoretical gap in peer feedback research. Prior work on learner engagement has predominantly applied a receiver-centric lens, derived from studies on teacher or automated feedback, which treats engagement primarily as the learner’s response to received input (Ellis, 2010; Han and Hyland, 2015; Zhang and Hyland, 2018). This perspective overlooks the active role of the feedback provider, resulting in what has been termed “observing only half of the conversation” (Philp and Duchesne, 2016). By systematically incorporating and theorizing learners’ engagement as feedback providers, this framework aligns analytical models with the fundamentally reciprocal and dialogic nature of peer feedback (Carless and Boud, 2018; Gibbs, 2014), thereby offering a more complete picture of the interaction.

The theoretical value of this framework is also reflected in its in-depth dialogue with current research on “feedback literacy.” In their learner-centered feedback literacy framework, Molloy et al. (2020) emphasize that feedback literacy should encompass two interrelated dimensions: the learner’s ability to understand, process, and use feedback information (receiver literacy), and the learner’s ability to provide constructive feedback, participate in feedback dialogue, and take responsibility within the feedback process (provider literacy). This perspective resonates strongly with the core logic of “dual-track parallelism” in the present framework.

This integration advances learner engagement theory in several key aspects. First, it shifts the research focus from a static analysis of learning outcomes to a dynamic, process-oriented examination. While previous studies have effectively measured gains in proficiency, they less often uncover the underlying mechanisms. This framework directs attention to the real-time, socio-cognitive process of feedback (Swain, 2006), enabling researchers to trace the sequences of reception, processing, negotiation, and revision, along with the accompanying behavioral and affective dynamics.

Second, the framework serves as a vital bridge between broad learning theories and observable classroom practice. Macro-level theories like sociocultural theory emphasize concepts such as scaffolding and meaning negotiation (Vygotsky, 1978), yet these can remain abstract. This model operationalizes these concepts into observable, role-specific behaviors and cognitive indicators. For instance, the act of providing scaffolded feedback becomes visible in a provider’s deep analysis and constructive suggestions (Track A), while the utilization of scaffolding is evident in a receiver’s active response and integration (Track B) (Nicol, 2010; Yu and Hu, 2017).

Third, it drives the contextual refinement of general engagement theory. The classic cognitive-behavioral-affective model (Fredricks et al., 2004) provides a universal foundation but may not capture the nuances of specific activities. This framework adapts and contextualizes these general dimensions for the unique environment of second language oral peer feedback. It demonstrates how engagement manifests differently across the provider and receiver roles and incorporates context-specific elements such as immediacy, multimodality, and real-time negotiation, setting a precedent for similar contextual adaptations in other learning domains.

Finally, the framework offers an integrated perspective on second language oral development, reconciling often-divergent cognitive and social research traditions. It theoretically unites the cognitive-metacognitive processes associated with processing feedback (Track B) with the social-interactive processes of providing feedback and engaging in negotiation (Track A and their interplay) (Swain, 2006; Yang and Carless, 2022). It posits that speaking proficiency develops through the sustained dialogue between these integrated pathways, where sociocognitive acts and individual cognitive work continuously inform each other.

Beyond its theoretical contributions, the framework carries important methodological implications. It provides a structured analytical map that highlights key variables and their relationships, guiding the design of tailored measurement tools—such as coding schemes, stimulated recall protocols, or multimodal discourse analysis—to fully capture the dual-track, interactive nature of engagement in future empirical research.

### 4.2 Practical implications for second language oral teaching

1. Instructional guidance: design and training

The dual-role engagement framework offers concrete guidance for enhancing peer feedback in L2 oral classrooms by translating its theoretical structure into practice. It advocates for tasks that engage both providers and receivers. For example, a complete cycle might include initial oral performance, structured feedback submission, recorded negotiation, revised performance, and provider reflections. Assessment should therefore become multidimensional, evaluating not only final oral improvement but also feedback quality, negotiation depth, and emotional experiences.

Training must explicitly address both roles. For providers, this includes cultivating in-depth analysis, application of criteria, constructive suggestion-making, empathy, and responsibility. For receivers, training should develop proactive questioning, critical evaluation of feedback, and openness. Cultural factors also matter: learners from face-saving cultures may use more euphemisms or experience heightened anxiety as providers (Track A) and defensiveness as receivers (Track B). Mode differences (online asynchronous vs. offline real-time) can further reshape interaction rhythms and emotional cues, requiring educator sensitivity in activity design. Practical implementation can draw on Alemdag and Yildirim’s (2022) scaffolded online peer assessment environment, which improved feedback depth and usability, and Brooks et al.’s (2021) finding that student-centered feedback enhances writing achievement and feedback literacy, both supporting structured dual-role training.

Training strategies should also be differentiated by proficiency. Beginners benefit from structured tools (e.g., checklists, templates) and form-focused exercises to reduce cognitive load and build confidence. Intermediate and advanced learners can be guided toward discourse, pragmatics, and interaction strategies, and encouraged to engage in more open meaning negotiation. Such tiered training equips learners to fulfil dual roles competently, maximizing engagement and learning outcomes.

1. The transformation of the teacher’s role

Within this framework, the teacher’s role shifts from being the primary feedback provider to becoming a designer and facilitator of the peer feedback ecosystem. Key responsibilities include fostering a supportive and trusting environment to encourage positive affective engagement; providing clear tools such as checklists and phrase banks to support cognitive and behavioral engagement; and monitoring peer interactions to intervene promptly when necessary. Such mediation acts as “scaffolding” that strengthens the dual-track interactive mechanism—for instance, by modeling how to deliver constructive criticism or pose clarifying questions.

1. Implementation case: structured task cycle and measurement methods

Based on the dual-role engagement framework, this paper proposes a practical teaching example, the “Structured Oral Task Cycle.” This cycle consists of five consecutive stages: learners first complete and record an initial oral output; then, based on a structured checklist, they provide written feedback to their peers, thereby stimulating and demonstrating cognitive and behavioral engagement within Track A (Provider Track); subsequently, both parties engage in oral negotiation based on the written feedback, simultaneously activating interactive behaviors in Track A and proactive responses in Track B (Processor Track); afterwards, learners integrate the feedback to produce a revised oral output, reflecting Track B’s processes of integration and output; finally, the teacher may offer summative comments as a form of advanced scaffolding. This cycle can be repeated throughout a course (e.g., over an academic year), enabling the longitudinal tracking of developments in dual-role engagement. To illustrate the framework’s practical application, consider a two-session peer feedback training workshop. In Session 1 (Feedback Providers), students analyze a sample oral narrative using a structured checklist. They practice cognitive engagement by diagnosing strengths/weaknesses, behavioral engagement by writing constructive comments, and affective engagement by delivering feedback empathetically. Session 2 (Feedback Receivers/Processors) uses the same narrative with pre-written comments. Students practice cognitive engagement by evaluating comment usefulness, behavioral engagement by formulating clarification questions during simulated negotiations, and affective engagement by maintaining openness to critique. After the workshop, students complete a real feedback cycle. Their recorded negotiations and revisions are analyzed using a simple coding scheme (e.g., identifying “deep analysis” in Track A, “active questioning” in Track B). This process demonstrates how the framework translates abstract concepts into observable teaching practices that foster meaningful peer interaction.

In addition to structured tasks, technology offers new ways to support dual-role engagement and feedback literacy. Wood (2022) found that online platforms enabling dialogic peer feedback can improve feedback uptake and literacy. For L2 oral contexts, tools like video recording and discussion forums help learners analyze oral performances, strengthening both providing and processing feedback. Cross-cultural considerations are also important. Rovagnati et al. (2022) showed that learners’ backgrounds influence feedback practices. Thus, teachers should integrate technology and cultural awareness to create inclusive environments that foster dual-role engagement and feedback literacy across diverse learners.

Beyond offering a theoretical perspective, this framework specifies concrete pathways for measuring dual-role engagement in empirical research. For the provider track (Track A), cognitive engagement can be assessed through diagnostic accuracy and metacognitive statements in written feedback (supplemented by stimulated recall); behavioral engagement via comment length, turn frequency, and discourse strategies; affective engagement through questionnaires on emotions like responsibility and anxiety, or content analysis of feedback tone. For the processor track (Track B), cognitive engagement is inferred from revision, feedback correspondence and stimulated recall; behavioral engagement from revision changes and negotiation responses; affective engagement via questionnaires on openness and trust, and analysis of verbal/non-verbal reactions during interaction. Multiple methods such as social network analysis, correlation, and longitudinal designs, can further capture feedback reciprocity, quality–uptake relationships, and dynamic engagement patterns. This operationalization transforms the framework into a structured teaching cycle with embedded data collection, enabling process-oriented assessment.

Looking ahead, the framework guides tool development for measuring dual-track engagement, exploring its links to learning outcomes, and examining how individual factors (e.g., proficiency, personality, culture) shape role-specific participation. Its core logic can also be adapted to other collaborative contexts (e.g., L2 writing peer review, online tasks), testing its explanatory boundaries.

### 4.3 Limitations and future empirical research directions

While the dual-role engagement framework proposed in this study offers a comprehensive theoretical lens for understanding peer feedback in L2 oral communication, it is important to acknowledge its current limitations. Most notably, this paper presents a conceptual model that has yet to be empirically validated. The framework is built upon a synthesis of existing literature and theoretical reasoning; therefore, its applicability, operationalizability, and explanatory power in real classroom contexts remain to be tested through systematic empirical investigation. Future research should prioritize the design and implementation of studies that can examine the framework’s validity and utility. For instance, to delve into the real-time cognitive decision-making and metacognitive processes of learners as they switch between the roles of feedback provider and receiver, studies could employ qualitative methods such as stimulated recall (Ahmed and Leider, 2024), using playback of interaction recordings to elicit participants’ reflective accounts, thereby uncovering deep cognitive mechanisms that quantitative data may not capture. Several promising directions emerge:

1. Instrument Development and Validation: Researchers could develop and validate measurement tools tailored to assess the multi-dimensional engagement constructs outlined in both Track A (provider) and Track B (receiver/processor). This could involve creating coding schemes for analyzing recorded peer interactions, designing self-report questionnaires or stimulated recall protocols to capture cognitive and affective engagement, and employing observational checklists for behavioral and social engagement.
2. Testing Framework Dynamics: Empirical studies are needed to investigate the proposed interactive mechanisms between the two tracks. Research questions could explore: How does the quality of engagement in the provider role influence the receiver’s subsequent engagement and revision outcomes? To what extent does the receiver’s response (e.g., uptake, negotiation) affect the provider’s sense of efficacy and future feedback behavior? Longitudinal or micro-genetic research designs would be particularly valuable for tracing these dynamic, reciprocal relationships over time.
3. Examining Contextual and Individual Factors: Future work should investigate how individual learner variables (e.g., language proficiency, personality traits, cultural backgrounds, motivation) and situational factors (e.g., task type, group composition, training methods, classroom culture) moderate learners’ engagement patterns in both roles. Such inquiries would help refine the framework and enhance its contextual sensitivity.
4. Comparative and Intervention Studies: Research could compare dual-role engagement across different feedback modalities (e.g., oral vs. written peer feedback) or sources (e.g., peer vs. teacher feedback). Additionally, intervention studies could assess the impact of specific pedagogical trainings—such as training focused on providing constructive feedback or on negotiating meaning—on enhancing engagement in one or both tracks, and subsequently on L2 oral development.
5. Multimodal Interaction Analysis: To deepen the dual-role framework empirically, future research should adopt multimodal interaction analysis as a key methodology. This involves developing coding schemes and using tools like ELAN to examine how learners coordinate gaze, gesture, facial expression, and prosody with verbal feedback in real time, managing face-threat, conveying empathy, signaling understanding, and regulating turn-taking. Following Ke (2025), comparative studies across face-to-face and video-conferencing contexts can further reveal how technological affordances shape multimodal engagement. Such work will strengthen the framework’s empirical grounding and explanatory power across diverse instructional settings.

Furthermore, the proposed framework may manifest differently across cultural contexts. Learners from collectivist or high-context cultures (e.g., East Asian) might exhibit distinct affective engagement patterns when providing feedback, such as greater use of mitigation strategies to maintain interpersonal harmony, and may experience heightened sensitivity when receiving criticism. Conversely, learners from individualistic or low-context cultures might engage more directly and assertively in both roles. These cultural variations could moderate the dynamics between Track A and Track B, influencing the effectiveness of peer feedback. Future research should systematically examine how cultural orientations shape dual-role engagement through cross-cultural comparative designs, testing the framework’s cross-cultural validity and informing culturally responsive pedagogical practices.

A critical limitation of this study is that the dual-role engagement framework was specifically constructed for and grounded in the unique characteristics of L2 oral peer feedback. Its core elements, such as the emphasis on immediacy, real-time negotiation, and multimodal cues (e.g., intonation, gestures), are intrinsically tied to oral interaction. Consequently, any attempt to apply or generalize this framework to other feedback contexts (e.g., L2 writing peer review, peer assessment in content courses, or online asynchronous discussions) would necessitate systematic contextual reconfiguration. For instance, in written feedback, the temporal dynamics of “providing” and “processing” are decoupled, the multimodality shifts to textual and graphic modes, and the negotiation process may be delayed or absent. Researchers or practitioners seeking to adapt the framework must therefore: (a) reconceptualize the role-specific engagement indicators to fit the new modality; (b) re-examine the interaction mechanisms between the two tracks given the altered temporal and communicative conditions; and (c) empirically validate the adapted framework before drawing conclusions. This caution ensures that the framework’s explanatory power remains robust within its intended domain while acknowledging the need for tailored adaptations elsewhere.

Addressing these limitations and pursuing these research directions will not only strengthen the empirical grounding of the dual-role engagement framework but also provide deeper insights into how to structure and facilitate peer feedback to maximize its potential for L2 oral learning.

## 5 Conclusion

This study systematically deconstructs the “single-track” perspective in peer feedback research by constructing a “dual-role engagement framework” for second language (L2) oral peer feedback. The core argument posits that peer feedback fundamentally involves learners engaging in social construction and cognitive processing through the dual roles of “provider” and “receiver/processor” in dialogue and negotiation. Existing learner engagement frameworks, developed primarily from teacher feedback paradigms, fail to capture this full process due to their neglect of the provider role. Integrating sociocultural theory and cooperative learning theory (Vygotsky, 1978; Slavin, 1980), this framework maps the three classic dimensions of cognition, behavior, and emotion onto two parallel tracks, “feedback provision” and “feedback processing,” and proposes a dynamic mutual shaping mechanism between them.

The framework marks a paradigm shift from viewing “feedback as a product” to understanding “feedback as collaborative practice.” It represents an epistemological shift that positions learners not as passive recipients but as active subjects with both “evaluator” and “constructor” identities. The “dual-track mutual shaping” mechanism reveals that learning effectiveness stems not only from feedback uptake but equally from the diagnostic thinking, expression organization, and emotional coordination experienced during feedback provision. The primary contribution lies in systematically integrating learners as feedback providers into the analytical framework for the first time, addressing a structural gap in existing theories of this reciprocal activity (Gibbs, 2014; Philp and Duchesne, 2016).

However, limitations must be acknowledged. First, this is a conceptual model requiring empirical validation through future research, including measurement tool development, classroom observations, and data analysis. Second, while originating from L2 oral contexts, its applicability to other collaborative learning scenarios requires careful adaptation and rigorous testing, making direct generalization inadvisable.

For classroom practice, a structured approach is recommended: designing interactive oral tasks requiring both performance and peer evaluation; providing clear tools and co-constructed criteria; developing students’ ability to give constructive feedback; facilitating structured cycles of output, feedback, negotiation, and revision; and assessing both feedback quality and evidence of processing, culminating in metacognitive debriefing (Carless and Boud, 2018; Nicol, 2010).

From a broader perspective, the framework reaffirms the integral relationship between “teaching” and “learning” in peer contexts, where providing and processing feedback constitute a complete developmental cycle. By emphasizing “role awareness” and “reciprocal relationship,” it calls for cultivating deeper socio-emotional capacities such as responsibility and empathy. In an era of online and blended education, the framework offers analytical dimensions for designing virtual collaborative activities, reminding us to consider how technology reconfigures role dynamics and engagement patterns among learners. Future research should extend this framework to investigate dual-role engagement across diverse settings, contributing to refined collaborative learning designs and realizing the full educational potential of peer interaction.

## Data Availability

The original contributions presented in the study are included in the article/supplementary material, further inquiries can be directed to the corresponding author/s.
